# Unveiling the multifaceted roles of anthocyanins: a review of their bioavailability, impacts on gut and system health, and industrial implications

**DOI:** 10.1016/j.crfs.2025.101137

**Published:** 2025-07-09

**Authors:** Fuqing Gao, Peiqing Yang, Wenxin Wang, Kewen Wang, Liang Zhao, Yongtao Wang, Xiaojun Liao

**Affiliations:** a College of Food Science and Nutritional Engineering, China Agricultural University, Beijing 100083, China; b National Engineering Research Center for Fruit and Vegetable Processing, Beijing, China; c Key Laboratory of Fruit and Vegetable Processing, Ministry of Agriculture and Rural Affairs, Beijing, China; d Beijing Key Laboratory for Food Non-thermal Processing, Beijing, China

**Keywords:** Anthocyanins, Gut microbiota, Metabolites, Bioavailability, Health benefits, Gut microecology

## Abstract

Anthocyanins, a class of flavonoids abundant in many fruits and vegetables, undergo complex catabolism, forming bioactive metabolites that profoundly affect a range of host physiological processes, including anti-inflammatory effects, modulation of oxidative stress, and modulation of the intestinal barrier. Despite this, the low bioavailability of anthocyanins presents challenges for their metabolic efficacy, health benefits, and therapeutic engagement *in vivo*. Therefore, the multifaceted roles of structurally diverse anthocyanins from different sources and the interplay between anthocyanin bioavailability and gut and systemic health were analyzed in depth. Additionally, we discuss how the dynamic gut microbial communities, i.e., growth/inhibition of certain microbial species, are regulated in diverse pathology models and the implications for anthocyanin metabolism and bioactivity in the context of gut and system health. A comprehensive analysis of the physicochemical factors affecting anthocyanin bioavailability and molecular mechanisms across disease models is undertaken. We further propose prospective techniques to enhance bioavailability, such as encapsulation, biotransformation, and omics-driven stratification of responder populations, as well as actionable insights for industry applications, including tailored food matrix design such as protein-polyphenol complexes, prebiotic co-delivery systems, and personalized nutrition strategies leveraging gut microbiota modulation. Ultimately, we advocate high-throughput analysis coupled with computational approaches such as AI-driven predictive modeling of anthocyanin-microbiota interactions to elucidate structure-activity relationships. These strategies are critical for determining whether observed health effects are attributable to anthocyanins per se or their synergistic/context-dependent actions, essential for enhancing bioavailability and therapeutic applications, thereby enabling rational dietary inclusion and therapeutic translation.

## Highlights

• Multifaceted roles of anthocyanins in diverse pathological settings are introduced.
• Detailed analysis of gut microbiota on anthocyanins bioavailability and health.
• Relationships on anthocyanin sources, prebiotics, and gut microecology are explored.
• High-throughput computational strategy for anthocyanin structure-activity assessment.

## Graphical abstract

**Figure:**
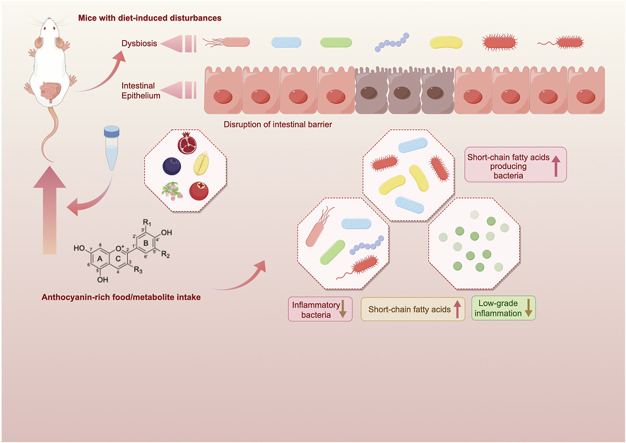

## 1 Introduction

**Fig. 1:**
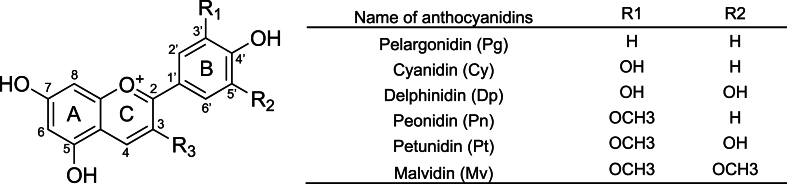
Schematic showing the general structure of six common anthocyanidins.

There is growing attention on the potential benefits of dietary flavonoids for human health and underlying mechanisms (Deng et al., 2025). Anthocyanins, a major subclass of flavonoid compounds responsible for the vibrant red, purple, and blue colors in many fruits, vegetables, and flowers (Alappat and Alappat, 2020; Khoo et al., 2017), garnered increasing attention for their potential health-promoting properties. However, the limited bioavailability of anthocyanins *in vivo* poses a significant challenge to fully realizing their therapeutic potential, necessitating a deeper understanding of the factors influencing their metabolism and bioactivity (Cai et al., 2022; Guo and Shahidi, 2024; He et al., 2022; Liang et al., 2023; Liu et al., 2024a; Liu et al., 2024; Saini et al., 2024; Verediano et al., 2021). Reviews of the literature concluded that the absence of homogeneity in anthocyanins bioavailability may fall into three distinct perspectives: the food components and processing conditions, the enzymes engaged in anthocyanins metabolism, and the anthocyanin-metabolizing gut microbiota (Di Lorenzo et al., 2021; Eker et al., 2019; Fernandes et al., 2014; Zheng et al., 2022). The most commonly studied anthocyanins are based on six anthocyanidins, as illustrated (Fig. 1), including Pelargonidin (Pg), Cyanidin (Cy), Delphinidin (Dp), Peonidin (Pn), Petunidin (Pt), and Malvidin (Mv), but approximately 700 anthocyanins are reported to be isolated from plants (Faria et al., 2014). The basic structure of anthocyanins is composed of an anthocyanidin aglycone and single or multiple glycosyl moieties (the most common glycosyl moieties are glucose, galactose, xylose, arabinose, and fructose) linked to hydroxyl groups (Cheng et al., 2014a), different anthocyanins differ in their glycoside and aglycone structures (Li et al., 2024a). Polyglycosylated anthocyanins, on the other hand, exhibit overall higher stability due to their complex structure than monoglycosylated ones under certain conditions, rendering them less susceptible to degradation and allowing for versatile applications (He et al., 2022; Li et al., 2024a). Anthocyanin glycosyl moieties are subject to acylation, whereby the hydroxyl groups of anthocyanin glycosides undergo esterification by aliphatic or aromatic acids (Jokioja et al., 2021). This acylation process is characterized by specific sites of modification, as well as the types and quantities of acyl groups introduced, and provides enhanced structural stability, thus lower bioavailability (Jokioja et al., 2021; Zhao et al., 2017a). Consequently, these variations significantly affect the chemical stability of anthocyanins both *in vitro* and *in vivo*, influencing their overall bioactivity and efficacy (Zhao et al., 2017a).

R_3_ represents different glycosyl moieties (glucose, galactose, etc.). The anthocyanin structure can be polyglycosylated, and the glycosyl moieties can also be acylated. Created by ChemDraw 23.1.1.

The health benefits of anthocyanins encompass a diverse range of protective effects, including antioxidant and anti-inflammatory activities (Chen et al., 2024a) as well as direct impact on cellular functions (Blesso, 2019), the management of obesity, the prevention of cognitive decline, and the safeguarding of essential organs such as the gastrointestinal tract, cardiovascular system, liver, and kidneys (Panchal et al., 2022). Anthocyanins were demonstrated to modulate gut microbiota populations, thereby improving diversity and the proportion of beneficial populations (Liang et al., 2023). The gut microbiota, a complex and dynamic community of microorganisms residing in the gastrointestinal tract, plays a pivotal role in the biotransformation of anthocyanins, significantly affecting their bioavailability and, in turn, their impact on host health (Liang et al., 2023; Liu et al., 2024a; Verediano et al., 2021). The gut microbiota also plays a critical role in modulating the biological effects associated with anthocyanins and their metabolites. This has consequently led to modifications in gut metabolites, specifically increased short-chain fatty acids (SCFAs) production (Liang et al., 2023). Furthermore, notable alterations in intestinal physiology were observed, characterized by a reduction in intestinal pH and permeability. These physiological changes also include an elevation in the number of goblet cells and tight junction proteins, as well as an enhancement in the length or height of intestinal villi (Verediano et al., 2021), thus shaping the gut microecology. Bioactive compounds belonging to the anthocyanins family, particularly cyanidin-3-O-glucoside (Cy3G), which is the predominant anthocyanin in many plant species (Khoo et al., 2017), along with its phenolic metabolites, were suggested to play a role in the prevention and progression of intestinal diseases (Cheng et al., 2023b). The study also revealed that oral administration of anthocyanins resulted in alterations to the gut microecology, encompassing the maintenance of anaerobic intestinal homeostasis, the promotion of secretory IgA and antimicrobial peptide secretion, the downregulation of cell motility, and the mobilization of mobile genetic elements associated with commensal bacteria (Liu et al., 2022). The intestinal mucosal immune system is central to the mechanisms through which these factors influence host gut diseases (Cheng et al., 2023b). Notably, specific microbial communities and their metabolites are influenced by anthocyanins; however, the mechanisms by which these products regulate signaling pathways and modulate physiological immune and metabolic responses remain poorly understood (Tan et al., 2019a).

This review addresses the critical gaps by synthesizing current literature to provide comprehensive insights into the specific gut microbial taxa involved in anthocyanins metabolism and the resulting metabolites; the impact of these metabolites on gut barrier function, inflammation, and systemic health; and the influence of dietary factors and inter-individual variability on the anthocyanins-gut microbiota interaction. Furthermore, we discuss novel hypotheses regarding the potential for personalized nutrition strategies, based on individual gut microbiota profiles, to enhance anthocyanins’ bioavailability and maximize their health benefits. By exploring these critical areas, this review aims to advance the understanding of the anthocyanins-gut microbiota axis and its implications for improving human health through targeted dietary interventions.

## 2 Methodology

**Fig. 2:**
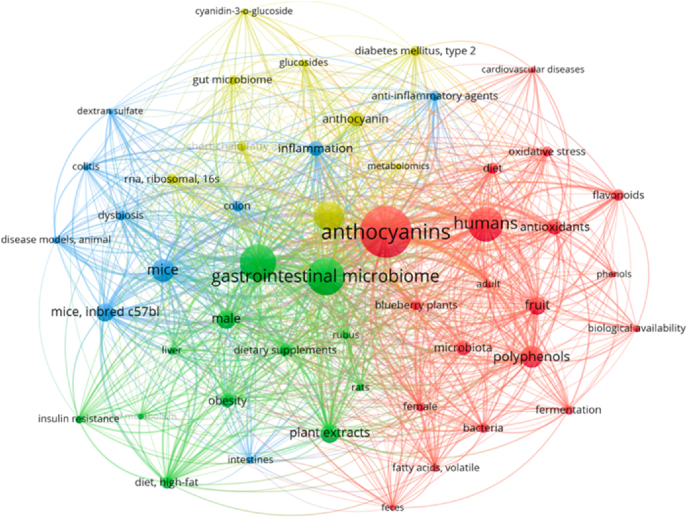
Top 50 high-frequency keywords (minimum number of occurrences of a keyword is 5) in the collected literature.

A comprehensive search is conducted across the available literature databases, based on the keywords ‘anthocyanins and microbiota’ or ‘anthocyanins and microbiome’, followed by the selection of articles describing the modulation of gut microbiota by structurally diverse anthocyanins, which facilitated subsequent classification and analysis on different anthocyanin sources in various disease types of intervention. Fig. 2 presents the top 50 high-frequency keywords extracted from the literature, analyzed using VOSviewer to quantify the co-occurrence link strength among terms. VOSviewer's algorithm calculated the total strength of co-occurrence links for each of the selected keywords. The visualization of keywords with the greatest total link strength provided critical insights that directly shaped the scope and structure of this review. The keyword analysis reveals a pronounced emphasis in the literature on the interplay between the gastrointestinal microbiome and anthocyanin interventions, particularly within the context of obesity and high-fat diet-induced conditions. This pointed to a need to synthesize findings on how anthocyanins modulate the gut microbiota in these prevalent metabolic disorders. Furthermore, the prominence of keywords related to anthocyanin sources—primarily fruits like blueberries and various plant extracts—guided our focus toward evaluating the impact of diverse anthocyanin sources on gut health. The occurrence of “antioxidant,” “anti-inflammatory,” and “health benefits” as high-frequency keywords highlighted the prevailing interest in anthocyanins' potential to mitigate oxidative stress and inflammation across various disease models. This observation led us to critically examine the mechanistic evidence supporting these claims. Finally, the keyword analysis demonstrates an increasing scientific interest in comprehending the connection of gut metabolites of anthocyanin-metabolizing microbiota (phenolic acids, SCFAs, etc.), inflammation status, and disease conditions, incorporating a metabolomic approach.

Building on these insights, this review provides a comprehensive overview of recent literature concerning the bioavailability of anthocyanins and their specific roles in the gut microbiota-health axis across various pathological models. We aim to elucidate strategies for optimizing the stability of anthocyanins and to identify key areas where further research is necessary to enhance our understanding of this dynamic field. By critically evaluating the current state of knowledge, this review seeks to offer new insights in the form of novel hypotheses, mechanistic understanding, and a discussion of controversies within this area of study, serving as a valuable resource for the academic community and sectors, including food, pharmaceutical, and cosmetic industries engaged in anthocyanin-rich bioactive food research and development.

## 3 Molecular mechanisms of anthocyanins in modulating the gut microbiota-health axis

Anthocyanins are a widely distributed class of dietary flavonoids recognized for their potential to exert beneficial effects in the context of chronic diseases linked to metabolic syndrome (Liu et al., 2024c). To address the impact of anthocyanins on gut microbiota structure, it is first necessary to understand their digestion behaviors (Liang et al., 2023). Dietary anthocyanins undergo a complex metabolic process following ingestion, either being absorbed as aglycones by the intestinal barriers in the upper gastrointestine tract or interacting with the gut microbiota to yield low-polymeric forms of anthocyanins or lower molecular weight phenolic metabolites, etc., with the assistance of endogenous and microbial enzymes (He et al., 2022; Rai and Tzima, 2021; Tian et al., 2019a). This results in the production of a multitude of excreted anthocyanin metabolites and catabolic products (Rai and Tzima, 2021; Tian et al., 2019a), and circulating metabolites and parental anthocyanins may enter into the bloodstream where they can reach peripheral organs and elicit bioactive effects in different pathologies (He et al., 2022; Teng and Chen, 2019).

Anthocyanins may modulate cellular antioxidant status and inflammatory equilibrium (Blesso, 2019; Ijinu et al., 2023). Although the effects of anthocyanins were demonstrated to be effective on several critical cells, including adipocytes, endothelial cells, immune cells, hepatocytes, and intestinal cells, there is currently a paucity of evidence regarding their impact on other cell types, such as hepatic stellate cells, skeletal muscle cells, and pancreatic beta cells, in the context of metabolic disorders related chronic diseases (Ijinu et al., 2023; Jiang et al., 2019a). The mechanisms by which these factors exert their effects on essential cellular functions and confer protection against chronic diseases will likely be intricate, not fully understood, and require further investigation (Blesso, 2019; Jiang et al., 2019a).

Imbalances in gut microbiota were implicated in the onset of low-grade inflammation, a persistent inflammatory state that significantly contributes to the development of various kinds of metabolic disorders (Liang et al., 2023), including cancer-related proinflammation, obesity, diabetes, neurodegeneration, cardiovascular diseases, aging, etc. Table 1, Table 2, Table 3 summarize a range of anthocyanin sources, from purified extracts to whole-food interventions. This variability is important to consider, as the food matrix can significantly influence anthocyanin bioavailability and metabolism. The versatility of anthocyanin structures enables them to participate in multiple pathological processes, either by directly influencing molecular pathways or modulating the pathological environment (He et al., 2022). While some studies utilized extracts from sources like purple sweet potato, bilberry, or black rice, providing a concentrated dose of specific anthocyanins, others incorporated whole foods such as blueberries or *Lycium ruthenicum*, which offer a more complex mixture of anthocyanins and other bioactive compounds. However, the difficulty in disentangling the effects of anthocyanins from those of other dietary compounds represents a significant challenge in this field of research (Grosso et al., 2022).

**Table 1:** Summary of studies (2019–2024) showing simultaneously anthocyanins' biological outcomes and effects on gut microbiota in inflammation and cancer models.

Type	Anthocyanins source	Animal model	Intervention	Biological outcomes	Effects on gut microbiota	References
Dextran sodium sulfate (DSS) induced colitis	Purple sweet potato anthocyanins (PSPA)	C57BL/6J male mice	10, 25 or 50 mg/kg per day of Pg3gal for 10 days	↓ IL-6, IL-1β, and TNF-α	↓ *Proteobacteria* and *Deferribacteres* ↑ Firmicutes, Bacteroidetes, and Verrucomicrobia	J. Chen et al. (2024)
*C. rodentium*-induced colitis	Blueberry malvidin-3-O-glucoside (Mv3G)	C3H/HeNCr male mice	4 mg/kg per day of Mv3G for 17 days	↑ Improved colonic hyperplasia and histopathological scores	↑ *Bifidobacterium animalis*	F. Liu et al. (2023)
DSS induced colitis	Mulberry anthocyanins (MAS)	C57BL/6J male mice	100 or 200 mg/kg of MAS for 17 days	↓ Disease activity index (DAI), colon shortening, tissue damage, inflammatory response and oxidative stress ↑ Intestinal tight junction (TJ) proteins (ZO-1, occludin and claudin-3)	↓ *Escherichia*-*Shigella ↑ Akkermansia, Muribaculaceae and Allobaculum*	Mo et al. (2022)
DSS induced colitis	Purple sweet potato anthocyanin extract (PSPAE)	C57BL/6J male mice	20 mg/kg PSPAE	↑ Intestinal barrier integrity	↓ Gammaproteobacteria and *Helicobacter* ↑ *Bifidobacterium* and *Lactobacillus*	Mu et al. (2021)
DSS induced colitis	AIN-93M diet containing Mv3G	C57BL/6J male mice	24 mg/kg per day of Mv3G for 50 days	↑ Improved histopathological scores, IL-10 and microbial interactions	↓ Pathogenic bacteria, such as *Ruminococcus gnavus*	F. Liu et al. (2019)
MC38 tumor	Bilberry anthocyanin extract (BAE)	C57BL/6 female mice	156 μg ACN (About 9–12 mg/kg) per day for 20 days	↑ Anti-tumor efficiency of the PD-L1 antibody and effective microbial diversity	*↑* Clostridia and *Lactobacillus johnsonii*	L. Wang et al. (2020)
Polystyrene-induced colonic inflammation	Red bayberry cyanidin-3-O-glucoside (Cy3G)	C57BL/6 mice	150 mg/kg of Cy3G for 6 weeks	↓ IL-6, IL-1β, and TNF-α; p-NF-κB, iNOS and COX-2 ↑ IL-22, IL-10, and IL-4	↓ *Desulfovibrio*, *Norank_f_Oscillospiraceae*, *Helicobacter*, and *Lachnoclostridium* ↑ *Dubosiella*, *Akkermansia*, and *Alistipes*	Chen et al. (2024)
Alcoholic liver injury	Black rice Cy3G	C57BL/6J male mice	100 mg/kg per day of Cy3G for 7 weeks	↓ Liver lipids ↑ Improved histopathological scores, intestinal barrier integrity and liver function	↓ Bacteroides, *Blautia*, *Collinsella*, *Escherichia-Shigella*, *Enterococcus*, *Prevotella*, *Ruminococcus_gnavus_group*, *Methylobacterium-Methylorubrum*, *Romboutsia*, *Streptococcus*, *Bilophila* ↑ *Norank_f_Muribaculaceae*	Zhu et al. (2024)
Cyclophosphamide (Cy)-induced intestinal damage	Purple red rice bran anthocyanins (PRBA)	BALB/c female mice	50, 100 and 200 mg/kg of PRBA for 7 days	↓ Intestinal barrier damage, cytokines, endotoxin (ET) and lipopolysaccharide binding protein (LBP) in serum ↑ TJ proteins, NF-κB pathway proteins, and short-chain fatty acids (SCFAs)	↓ *Shigella* ↑ Lachnospiraceae, Bacteroidaceae, Ruminococcaceae	T. Chen et al. (2022)
3-Chloro-1,2-propanediol (3-MCPD) induced intestinal injury	Black soybean coats Cy3G	Wistar male rat	500 or 1000 mg/kg Cy3G for 8 weeks	↑ Improved histopathological scores of intestinal villus, microvillus and mucosa	↑ Lachnospiraceae_NK4A136_group and Actinobacteria	Chen et al. (2019)

**Table 2:** Summary of studies (2019–2024) showing simultaneously anthocyanins' biological outcomes and effects on gut microbiota in obesity models.

Type	Anthocyanins source	Animal model	Intervention	Biological outcomes	Effects on gut microbiota	References
High-fat diet (HFD)-induced obesity	Blueberry anthocyanins	C57BL/6J male mice	615 mg/kg per day of ACN for 10 weeks	↓ Liver lipid droplet accumulation and injury	↑ Beneficial bacteria and acetic acid-producing bacteria, Coriobacteriales (Order)	Cheng et al. (2024)
HFD-induced hypercholesterolemia	*Lycium ruthenicum* Murray anthocyanins	ApoE−/− mice	15 mg/ml ACN solution for 16 weeks	↓ Serum concentrations of total cholesterol (TC) and low-density lipoprotein cholesterol levels (LDL-C), conjugated/unconjugated bile acids (BAs) ratio in feces, lipid accumulation in liver and adipose tissues	↓ *Ileibacterium* (Genus), *Helicobacter* (Genus), *Rikenellaceae_RC9_gut_group* (Genus), *Blautia* (Genus), *Odoribacter* (Genus), and *Colidextribacter* (Genus) *↑ Bifidobacterium* (Genus) and *Allobaculum* (Genus)	Zhang et al. (2023)
HFD-induced obesity	Blueberry, blackberry anthocyanin extracts (VA and RA)	C57BL/6J male mice	100 mg/kg VA and RA for 16 weeks	↓ Body weight gain, fat accumulation, liver damage, and inflammation ↑ Glucose and lipid metabolism	↓ Actinobacteria (Phylum), *Allobaculum* (Genus) and *Bifidobacterium* (Genus) ↑ Bacteroidetes (Phylum), *Prevotella* (Genus) and *Oscillospira* (Genus)	Du et al. (2023)
HFD-induced obesity	*Lycium ruthenicum* Murray Petunidin-3-O-[rhamnopyranosyl-(trans-p-coumaroyl)]-5-O-(β-D-glucopyranoside)	C57BL/6J male mice	100 mg/kg P3G for 12 weeks	↓ Body weight gain, fat accumulation, and liver steatosis	↓ Firmicutes (Phylum) and *Lactobacillus* (Genus)	P. Liu et al. (2022)
High-fat/cholesterol diet (HCD)-induced obesity	Purple sweet potato anthocyanin extract (PSPAE)	C57BL/6J male mice	3, 6, and 9 g PSPAE/kg diet for 12 weeks	↓ Body weight gain, TC, serum triglyceride (TG), serum activities of glutathione peroxidase, superoxide dismutase, catalase, serum contents of malondialdehyde, lipopolysaccharides ↑ Caecal total SCFAs	↑ *Akkermansia* (Genus), *Bifidobacterium* (Genus), and *Lactobacillus* (Genus)	D. Liu et al. (2022)
HFD-induced obesity	Pomegranate peel anthocyanins (PPA)	C57BL/6J male mice	150 mg/kg PPA for 15 weeks	↓ Body weight gain, steatosis scores and insulin resistance index	↓ Firmicutes (Phylum)*,* Proteobacteria (Phylum)*, Escherichia-Shigella* (Genus) ↑ Bacteroidetes (Phylum), Verrucomicrobia (Phylum), *Akkermansia*, *Parabacteroides*, *Anaerotruncus*, *Lachnoclostridium* (Genus)	Song et al. (2022)
HFD-induced obesity	*Lycium ruthenicum* Murray anthocyanins	C57BL/6J male mice	0.8 % crude extract of ACN for 14 weeks	↓ Body weight gain, lipid accumulation in liver and white adipose tissue, serum TC and LDL-C	↓ Firmicutes/Bacteroidetes ratio (Phylum), *Faecalibaculum* (Genus) ↑ *Akkermansia* (Genus)	N. Li et al. (2022)
HFD-induced obesity	*Lycium ruthenicum* Murray anthocyanins	C57BL/6J male mice	50, 100, or 200 mg/kg ACNs for 12 weeks	↓ Endotoxin (i.e., lipopolysaccharide) levels, weight gain ↑ SCFAs content	*↓* Endotoxin-producing bacteria (e.g., Desulfovibrionaceae (Family) and *Helicobacter* (Genus)) *↑* SCFA-producing bacteria (e.g., *Ruminococcaceae* (Family), *Muribaculaceae* (Family), *Akkermansia*, *Ruminococcaceae_UCG-014*, and *Bacteroides* (Genus))	B. Tian et al. (2021)
HFD-induced obesity	Blackcurrant anthocyanins (BCA)	C57BL/6J male mice	150 mg/kg BCA for 14 weeks	↓ Hyperlipemia and hepatic steatosis ↑ Hepatic lipid metabolism	↑ *Akkermansia muciniphila* (Species)	Song et al. (2021a)
HFD-induced obesity	Blueberry, cranberry anthocyanin extracts	C57BL/6J male mice	1 % or 2 % blueberry or cranberry extracts for 24 weeks	↓ Plasma lipopolysaccharide	*↓* Rikenellaceae (Family) and *Rikenella* (Genus) and ↑ *Lachnoclostridium*, *Roseburia*, and *Clostridium_innocuum_group* (Genus)	J. Liu et al. (2021)
HFD-induced hyperlipidemia	*Aronia melanocarpa* (AAM) anthocyanins	C57BL/6J male mice	25, 100 mg/kg of AAM for 12 weeks	↑ Anti-adipogenic activities, AMP-activated protein kinase (AMPK) signaling pathway	↓ *Proteobacteria*, *Bilophila*, *Lachnobacterium*, and *Helicobacter* (Genus) ↑ Firmicutes/Bacteroidetes ratio (Phylum), *Dehalobacterium* (Genus)	Chen et al. (2021)
HFD-induced obesity	Black rice anthocyanins (BRAN)	C57BL/6J male mice	BRAN supplement for 14 weeks	↓ Body weight gain, TG, total cholesterol, steatosis scores and insulin resistance index	↓ Firmicutes/Bacteroidetes ratio (Phylum)	＀＀Song et al. (2021b)
High-fat and the cholesterol diet (HCD)-induced hypercholesterolemia	Black rice anthocyanin extract (BRAE)	C57BL/6J male mice	18, 36, 72 mg/kg per day of BRAE for 12 weeks	↓ Body weight gain, TG, TC, non-high-density lipoprotein cholesterol levels (non-HDL-C), fecal sterols excretion and caecal SCFAs	↓ Firmicutes/Bacteroidetes ratio (Phylum), *Clostridium* (Genus) and *Desulfovibrio* (Genus) ↑ *Allobaculum*, *Bacteroides*, *Lactobacillus*, *Bifidobacterium*, *Parabacteroides*, *Oscillospira*, *Akkermansia*, *Ruminococcus*, and *Butyricimonas* (Genus)	H. Wang et al. (2020)
Western diet (WD)-induced metabolic dysfunction-associated steatotic liver disease (MASLD)	Bilberry anthocyanin extract	C57BL/6N male mice	2 % ACN for 18 weeks	↓ Serum levels of aspartate aminotransferase (AST), alanine aminotransferase (ALT), LDL-C, fat content in liver, 2-thiobarbituric acid reactive substances (TBARS) and α-smooth muscle actin (α-SMA)	↓ Firmicutes/Bacteroidetes ratio (Phylum) ↑ *Akkermansia* (Genus) and *Parabacteroides* (Genus)	Nakano et al. (2020)

**Table 3:** Summary of studies (2019–2024) showing simultaneously anthocyanins' biological outcomes and effects on gut microbiota in diabetes models.

Type	Anthocyanins source	Animal model	Intervention	Biological outcomes	Effects on gut microbiota	References
Type 2 diabetes mellitus (T2DM)	Purple sweet potato anthocyanins (PSPA)	Kunming male mice	227.5, 455, or 910 mg/kg PSPA for 10 days	↓ Fasting blood glucose (FBG), glycosylated hemoglobin ↑ Blood glutathione	↓ Firmicutes/Bacteroidetes ratio, nine bacterial genera, including *Staphylococcus ↑ Lachnospiraceae_Clostridium, Butyricimonas, and Akkermansia*	Mi et al. (2024)
T2DM	Red bayberry-derived Cy3G	db/db male mice	150 mg/kg per day of Cy3G for 6 weeks	↓ Glucose level ↑ Glucagon-like peptide (GLP-1), short-chain fatty acids and insulin	↓ Firmicutes/Bacteroidetes ratio, *Lactobacillus*, *Lachnospiraceae_NK4A163_group*, *Norank_f_norank _o_Clostridia_UCG-014*, and *Bacteroides* ↑ Prevotellaceae_NK3B31_group, Prevotellaceae_UCG-001 and *Helicobacter*	Ye et al. (2023)
T2DM	Black bean and black rice anthocyanin extract	Rats	Black bean husk and black rice anthocyanin extracts for 4 weeks	↓ Liver damage ↑ Improved blood glucose, insulin resistance, serum oxidative stress, lipid metabolism and inflammatory cytokines levels, AMPK, PI3K, and AKT	↑ Short-chain fatty acid (SCFA) producing bacteria (e.g., *Akkermansia* spp., *Phascolarctobacterium* spp., *Bacteroides* spp., and *Coprococcus* spp.)	Sun et al. (2022)
T2DM	Nonacylated anthocyanins from bilberries (NAAB) or acylated anthocyanins from purple potatoes (AAPP)	ZDF male rats (fa/fa)	25 or 50 mg/kg per day of NAAB or AAPP for 8 weeks	↑ Caecal sugar levels	*↓ Parabacteroides* spp. ↑ Peptostreptococcaceae sp.	K. Chen et al. (2022)
T2DM	Wild raspberry pelargonidin-3-O-glucoside (Pg3G)	Db/db male mice	150 mg/kg per day of Pg3G for 8 weeks	↑ Glucose tolerance, insulin sensitivity, autophagy and intestinal barrier integrity	↓ Firmicutes/Bacteroidetes ratio ↑ *Prevotella*	Su et al. (2020)
High fat-high sucrose (HFHS) diet-induced insulin-resistant	Polyphenols Cy3G extract	C57BL/6J male mice	7.2 mg/kg per day of Cy3G for 11 weeks	↓ Glucose, lipids, insulin resistance and inflammatory markers	↓ Firmicutes/Bacteroidetes ratio ↑ Muriculaceae	Huang et al. (2020)

### 3.1 Inflammation and cancer

Emerging evidence positions anthocyanins as promising candidates for preventing and treating intestinal complications (Fig. S1), including inflammatory bowel disease (IBD), irritable bowel syndrome, and colorectal cancer (Liu et al., 2024a). While the observed protective effects are frequently attributed to improvements in the intestinal epithelial barrier, immune modulation, antioxidant and anti-inflammatory properties, and alterations in gut microbiota metabolism (Liu et al., 2024a), a critical examination reveals a need for more nuanced mechanistic insights. A summary of studies (2019–2024) has attempted to show relationships between anthocyanins' biological outcomes and effects on gut microbiota in inflammation and cancer models (Table 1); however, the precise mechanisms and the extent to which these effects are directly attributable to anthocyanins versus their metabolites remain under investigation, representing a crucial area for future research.

Anthocyanin extracts, such as those from purple sweet potatoes and mulberry, were demonstrated the ability to alleviate dextran sodium sulfate (DSS)-induced chronic colitis by inhibiting pyroptosis in intestinal epithelial cells, thereby maintaining the intestinal barrier, improving colonic architecture, and modulating gut microbiota composition (Chen et al., 2024b; Liu et al., 2024a; Mo et al., 2022; Mu et al., 2021). These modulations are characterized by an increase in the Firmicutes and Verrucomicrobia phyla (represented by *Lactobacillus* and *Akkermansia* genera, respectively) and a decrease in the Proteobacteria phylum (represented by *Helicobacter*, *Escherichia*, and *Shigella* genera) (Chen et al., 2024b; Mo et al., 2022; Mu et al., 2021). However, these findings must be interpreted with caution, as DSS-induced colitis models do not fully replicate the complexities of human IBD, and the observed shifts in microbiota may not directly translate to therapeutic benefits in humans. A critical evaluation of the specific bacterial species affected and their functional roles in the context of IBD is essential (Chen et al., 2024b). Among the anthocyanin family, Cy3G may represent a promising supplement for alleviating IBD, while its metabolites may serve as critical leading compounds for developing new therapeutic agents (Frountzas et al., 2023). While the potential effects of this compound were documented across various *in vitro* and *in vivo* contexts, further preclinical and clinical investigations are essential to confirm its potency (Frountzas et al., 2023). Moreover, the bioavailability of Cy3G and its metabolites *in vivo*, which are affected by pH, light, and enzymatic modifications, influences the therapeutic potential of the compound (Li et al., 2023b; Rashwan et al., 2023; Samota et al., 2022). The consumption of malvidin-3-O-glucoside (Mv3G), another bioactive compound within the anthocyanin family, has been shown to improve both the histopathological scores and the evenness of microbial communities (Liu et al., 2023d), and restore the Firmicutes/Bacteroidetes ratio in the colon mucosa of colitis mice (Liu et al., 2019e). Furthermore, Mv3G reduced the abundance of pathogenic bacteria and tested microbial challenges in the study, such as *Ruminococcus gnavus* and *Citrobacter rodentium,* via a pronounced modulatory effect on inflammatory signaling pathways, microbial co-occurrence patterns, and gut metabolites (Liu et al., 2019e; Liu et al., 2023d). Specifically, Mv3G reversed bodyweight loss from *C. rodentium* infection and improved colon health by increasing Hace1 expression, impacting TGF-β signaling, and enhancing the probiotic *Bifidobacterium animalis* (Liu et al., 2023d). Additionally, Mv3G modulates microbial dysbiosis and mediates host- and microbiota-derived metabolites. It's critical to acknowledge that the gut microbiota's impact on anthocyanin metabolism is bidirectional: anthocyanins can modulate the gut microbiota, and in turn, the gut microbiota can transform anthocyanins into various metabolites (Morais et al., 2016). As a bioactive compound, Mv3G influences the gut microbiota through a mechanism distinct from that of whole blueberries (Liu et al., 2019e), while whole blueberries are recognized as one of the most effective functional fruits, largely due to their high content of anthocyanins and polyphenols, which play significant roles in the prevention of chronic diseases (Ma et al., 2018a). Such synergistic effects, or lack thereof, in combining anthocyanins with other dietary components, should be carefully considered. Interestingly, the administration of malvidin-3-O-galactoside (galactose instead of glucose at the R_3_ position of the anthocyanidin) was observed to enhance the physical and immune barrier function of the colonic mucosa in a mouse colitis model, with this effect being mediated by the Notch signaling pathway (Zhang et al., 2024a). Further research into various contributions of the effect of the substitution between monosaccharides, factors that influence and modulate the colonic mucosal barrier, is warranted to identify novel strategies for preventing and treating these disorders, thus maintaining digestive tract homeostasis and systemic metabolic equilibrium (Zhang et al., 2024a). Given that the Notch signaling pathway is crucial to governing diverse cell processes, including cell-fate determination, differentiation, proliferation, and apoptosis, more mechanistic studies into how malvidin-3-O-galactoside interacts with this signaling pathway to promote a functional colonic barrier are needed (Jiang et al., 2022b; Ning et al., 2024; Pu et al., 2021).

Anthocyanins and their metabolites produced by gut microbiota possess significant potential as functional foods and therapeutic agents for the modulation of aging processes and the management of age-related diseases (Hair et al., 2021; B. Wang et al., 2024). Nevertheless, the molecular mechanism through which anthocyanins scavenge senescent, aging endothelial cells is highly intricate and not yet fully elucidated (Dong et al., 2022; Hair et al., 2021; Trinei et al., 2022). While studies suggest the activation of pathways like phosphoinositide 3-kinase/protein kinase B/endothelial nitric oxide synthases and silent information regulator 1, or inhibition of nuclear factor kappa B, Bax, or P38 mitogen-activated protein kinase pathways (Bashllari et al., 2023; Zhao et al., 2024b), a comprehensive, systems-level understanding is still lacking.

The protective effects of dietary Cy3G against microplastics and polystyrene exposure and the regulation of specific gut bacteria, their metabolites, and functional pathways in response to xenobiotics in the amelioration of colonic inflammation were highlighted (Chen et al., 2022c; Chen et al., 2024d). Dietary Cy3G appears to exert its anti-inflammatory effects by modulating gut microbiota, which in turn influences tryptophan and bile acid metabolism (Chen et al., 2024d). Cy3G enriches bacterial genes involved in tryptophan and bile acid metabolic pathways which leads to the upregulation of key metabolites such as shikimate, L-tryptophan, and specific bile acids, suggesting that Cy3G promotes a shift towards metabolic profiles that actively dampen colonic inflammation and are associated with the increase of Verrucomicrobia phylum represented by *Akkermansia* genus and decrease of Proteobacteria phylum represented by *Helicobacter* and *Desulfovibrio* genus (Chen et al., 2024d). However, it's important to note that the study focused on the effects of a single anthocyanin (Cy3G) and a specific type of microplastic (polystyrene). The impact of other anthocyanins and microplastics, as well as their combinations, may differ. In particular, the complex interactions between gut microbiota, tryptophan, and bile acid metabolism influenced by Cy3G supplementation were not elucidated (Chen et al., 2024). Similarly, the protective effect of Cy3G against 3-chloro-1,2-propanediol-induced gut dysbiosis was also reported (Chen et al., 2019). In addition, Cy3G has been demonstrated to alleviate alcohol-induced liver injury through the modulation of gut microbiota and metabolites, indicating its potential for preventing and managing alcoholic liver disease as a functional supplement (Zhu et al., 2024). The purple-red rice bran anthocyanins were demonstrated to enhance intestinal barrier function and restore gut microbiota balance disrupted by cyclophosphamide treatment. This suggests a promising avenue for the utilization of anthocyanins derived from dark-colored cereals in nutritional applications (Chen et al., 2022). While these findings suggest a protective role against chemotherapy-induced gut dysbiosis, the specific mechanisms by which anthocyanins modulate the gut microbiota and influence immune responses in this context require further investigation.

The antitumor effects of anthocyanins have been demonstrated to involve multiple genes and signaling pathways, which have been associated with many processes, including inflammation, oxidative stress, cell proliferation, apoptosis, cell invasion, angiogenesis, metastasis, and the modification of the gut microbiota (Nascimento et al., 2023). For example, anthocyanins inhibit the formation and growth of colorectal cancer in azoxymethane/DSS-treated BALB/c mice (Lippert et al., 2017). The potential of orally administered bilberry anthocyanin extracts to enhance the anti-tumor efficacy of PD-L1 antibody and increase Firmicutes phylum in the experimental mouse MC38 tumor model is also highlighted (Wang et al., 2020a). Notably, research has demonstrated that certain anthocyanins can reach the colon without undergoing degradation, thereby being subjected to metabolic processes by the resident microbiota (Nascimento et al., 2023). The modulation of gut metabolism and the release of antiproliferative metabolites by the gut microbiota can positively influence the body's metabolic processes (Dharmawansa et al., 2020; Nascimento et al., 2023; Williamson and Clifford, 2017). These processes have the potential to reduce or eliminate dysbiosis, a condition that is often observed in individuals diagnosed with colorectal cancer (Dharmawansa et al., 2020; Nascimento et al., 2023; Williamson and Clifford, 2017). However, recent studies indicate that the relationship between dietary polyphenols and cancer management is complex and not always beneficial, such as antibiotic therapies (Karamanolis et al., 2024). More research is needed to fully understand the context-dependent effects of anthocyanins on cancer development and progression, such as the effects of antibiotics or disease stages.

### 3.2 Obesity-related disturbances

It is becoming increasingly evident that the inflammatory processes associated with obesity may trace their origin to gut dysfunction, including changes within the intestinal bacteria or alterations in the gut microbiota cohort (Jayarathne et al., 2019). The upstream and downstream of the leptin pathway, for example, have recently been suggested to be regulated by the activity of anthocyanins (Liu et al., 2024c). While adipose tissue contributes majorly to systemic disorders (Jayarathne et al., 2019), anthocyanins could regulate the hypertrophy of adipose tissue and the expression of leptin levels by mediating key transcription factors and inflammatory cytokines, including tumor necrosis factor-alpha, CCAAT/enhancer binding protein isoforms, peroxisome proliferator-activated receptor gamma, cAMP-response element binding protein, and sterol regulatory element-binding protein 1, and influence leptin sensitivity by promoting leptin receptor expression and downstream signaling through the Janus kinase 2/signal transducer and activator of transcription 3 and phosphoinositide 3-kinase/protein kinase B pathways (Liu et al., 2024c). Circulating leptin concentrations in healthy individuals typically range from 5 to 10 ng/mL; these levels can increase dramatically, reaching 50 ng/mL or higher in obese and diabetic individuals, contributing to leptin resistance (Liu et al., 2024c). While initial hypotheses centered on adipose tissue as the primary driver, emerging evidence suggests that gut dysbiosis may precede and exacerbate adipose tissue inflammation, creating a feed-forward cycle (Jamar et al., 2017). Upon absorption, anthocyanins were demonstrated to confer beneficial effects on skeletal muscle and adipose tissue by enhancing the signaling through the AMP-activated protein kinase pathway and restoring insulin sensitivity, which could represent a novel avenue for the dietary management of obesity and its related complications (Solverson, 2020). Given their anti-inflammatory properties, anthocyanins are hypothesized to alleviate obesity-related disturbances in gut microbiota, insulin resistance, oxidative stress, and inflammation. Consequently, they may serve as a valuable therapeutic intervention for obese individuals (Xie et al., 2018). The potential underlying mechanisms involved are the inhibition of lipid absorption, the modulation of lipid metabolism, the increase in energy expenditure and energy balance, the suppression of food intake, and gut microbiota regulation (Sivamaruthi et al., 2020; Xie et al., 2018). A summary of studies (2019–2024) was presented, showing simultaneously anthocyanins' biological outcomes and effects on gut microbiota in obesity models (Table 2).

**Fig. 3:**
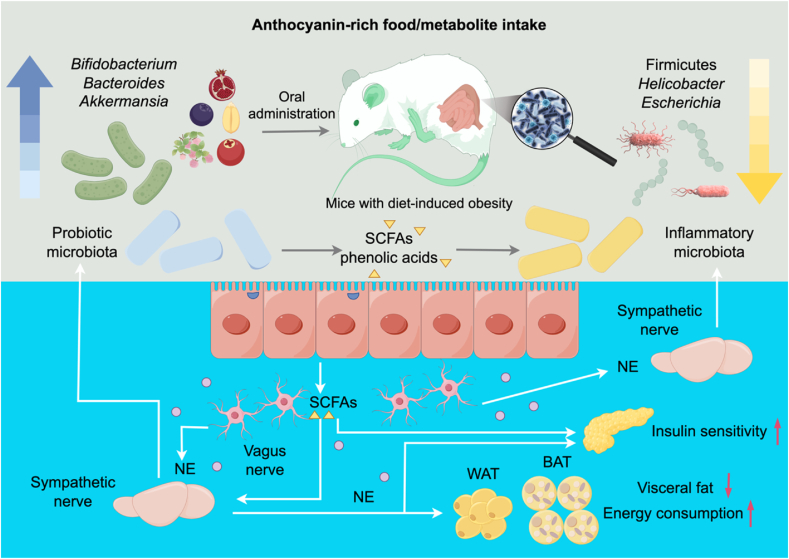
Schematic showing the potential biological outcomes associated with the intake of anthocyanin-rich foods and metabolites, emphasizing their impact on gut microbiota and pathways pertinent to obesity-related disturbances. The consumption of anthocyanin-rich foods and their metabolites typically increases the *Bifidobacterium*, *Bacteroides,* and *Akkermansia* genera, while decreasing the *Escherichia* and *Helicobacter* genera (Jayarathne et al., 2019). At the phylum level, there is a consistent observation of increased Bacteroidetes and decreased Firmicutes. The figure also highlights the anti-adipogenic signaling pathways modulated by gut-derived metabolites (Liu et al., 2022). Specifically, the gut metabolites produced by anthocyanin-metabolizing microbiota, including short-chain fatty acids (SCFAs) and phenolic acids, may exert a regulatory influence on gut microecology, host glucose, and lipid metabolism (Jamar et al., 2017; Sivamaruthi et al., 2020; Xie et al., 2018). This regulation is thought to occur through neuro-regulatory mechanisms mediated by norepinephrine (NE), which functions as a neurotransmitter influencing the energy expenditure of both the white adipose tissue (WAT) and brown adipose tissue (BAT) (Gao et al., 2022). Created by http://www.figdraw.com/#/.

Anthocyanins derived from dark-colored fruits, vegetables, and cereals (see Table 2: *Lycium ruthenium* Murray, blackcurrant, blueberry, blackberry, cranberry, pomegranate peel, purple sweet potato, and black rice, etc.) were demonstrated to alleviate hyperlipemia in mice induced by a high-fat diet or high-fat combined with the cholesterol diet. The data presented in Table 2 elucidate the specific effects on gut microbiota homeostasis, demonstrating a general enhancement in the populations of SCFAs-producing bacteria, represented by the *Bacteroides* and *Akkermansia* genus, and a reduction in the Firmicutes to Bacteroidetes ratio and Proteobacteria phylum and endotoxin-producing bacteria represented by the *Helicobacter* genus (Fig. 3). These effects were associated with the control of body weight, improvement of intestinal barrier function, modulation of lipid metabolism, as well as inflammation conditions (Du et al., 2023; Liu et al., 2021f; Liu et al., 2022; Song et al., 2022; Song et al., 2021b; Song et al., 2021a; Tian et al., 2021b; Wang et al., 2020b; Zhang et al., 2023b) and involve regulation of anti-adipogenic signaling pathways (Fig. 3), redox state related to gut dysbiosis (Liu et al., 2022), and inhibition of pancreatic lipase activity (Li et al., 2022c). It is plausible that the concentration of anthocyanins, alongside their unique structural profiles in each matrix, could differentially modulate gut microbiota, leading to variations in cellular stimulation and subsequent health outcomes. Specifically, variations in glycosylation patterns, hydroxylation, and acylation of anthocyanins may influence their interaction with gut microbiota, thus affecting the production of specific SCFAs and modulating the host's physiological response. For instance, acylation with p-coumaric acid or a second sugar moiety protected anthocyanins in the gut (Selma et al., 2009).

Further comparative analyses are needed to correlate anthocyanin content and structural diversity across different sources with specific changes in gut microbiota composition and their resultant impact on cellular pathways related to lipid metabolism and inflammation. This is an area ripe for exploration, moving beyond simply identifying which bacteria are altered to understanding how these bacteria are metabolizing different anthocyanin structures and what specific metabolites are being produced. The antiatherogenic effect of protocatechuic acid (PCA), the gut microbiota metabolite of Cy3G, is partially mediated by a defined miRNA-cholesterol efflux signaling cascade, which suggests that the gut microbiota metabolism of anthocyanins may represent a novel target for atherosclerosis prevention and treatment (Wang et al., 2012c). It has been postulated that anthocyanins and PCA could exert a beneficial effect on metabolic dysfunction-associated steatotic liver disease (MASLD) through the regulation of key metabolic pathways, including glucose and lipid metabolism, oxidative stress, inflammatory responses, gut microbiota homeostasis, and the modulation of gut metabolites (Gao et al., 2022). The initial hypothesis suggests that PCA may mitigate the severity of MASLD by enhancing energy expenditure in BAT (Gao et al., 2022).

### 3.3 Diabetes-related disturbances

The administration of anthocyanins has been demonstrated to effectively modulate fasting blood glucose levels and glycated hemoglobin while also influencing gut microbiota diversity and composition, along with other indicators pertinent to the pathogenesis of diabetes (Fig. S2), such as oxidative stress state, inflammatory cytokines levels, and lipid metabolism, etc. (Chen et al., 2022g; Sun et al., 2022). Moreover, higher anthocyanin dosages were associated with more favorable outcomes in the context of diabetes treatment (Chen et al., 2022g; Kozłowska and Nitsch-Osuch, 2024; Su et al., 2020). Despite the inconsistencies in the findings of multiple clinical trials, which may be attributed to factors such as the quantity ingested, the food matrix effect, and the “before or after a meal” timing, evidence suggested that anthocyanins may possess anti-diabetic properties (Oliveira et al., 2020). Table 3 presents a summary of studies (2019–2024) showcasing the biological outcomes of anthocyanins and their effects on gut microbiota in diabetes models.

The data presented in Table 3 indicate specific effects on gut microbiota homeostasis, including a general increase in SCFAs producers, particularly those belonging to the *Akkermansia* genus, alongside a decrease in the Firmicutes/Bacteroidetes ratio at the phylum level. While these trends are promising, it is crucial to acknowledge that the Firmicutes/Bacteroidetes ratio is an oversimplified marker of gut health, and its direct correlation with disease states is debated (Gu et al., 2023; Su et al., 2020). In particular, the bioactive compound Cy3G within the anthocyanin family may serve as a potential prebiotic that is capable of mitigating the negative metabolic consequences, inflammatory responses, and gut microbial dysbiosis associated with high fat-high sucrose diet and has been demonstrated to contribute to the metabolic benefits conferred by Saskatoon berry powder (Huang et al., 2020). In addition, the mechanistic explanation of Cy3G acts on Type 2 diabetes mellitus (T2DM) involves the modulation of gut bacteria metabolic activities, leading to increased intestinal levels of key metabolites, notably SCFAs (Ye et al., 2023). SCFAs such as butyrate, propionate, and acetate, not only serve as an energy source for the colonic epithelium but also engage host signaling pathways that modulate both appetite and inflammation (Ye et al., 2023). These SCFAs stimulate the secretion of glucagon-like peptide-1 (GLP-1), an incretin hormone known to enhance insulin secretion and improve glucose homeostasis (Ye et al., 2023; Yoon et al., 2021). SCFAs can trigger GLP-1 secretion, thereby improving insulin secretion. Furthermore, propionate, a SCFA, is a precursor for hepatic glucose production (Ye et al., 2023). The administration of Pg3G led to a significant enhancement in both glucose tolerance and insulin sensitivity, accompanied by the induction of autophagy (Su et al., 2020), thus alleviating diabetes conditions. Autophagy, a cellular self-degradative process, may play a critical role in mediating the beneficial effects of Pg3G on diabetes-related outcomes (Su et al., 2020). Moreover, Pg3G not only altered gut microbiota composition, characterized by increased prevalence of the *Prevotella* genus and an elevated Bacteroidetes/Firmicutes ratio, but also reinforced intestinal barrier integrity (Su et al., 2020). The increased abundance of *Prevotella* may be attributed to its integral role in carbohydrate utilization and its ability to metabolize glucose (Kovatcheva-Datchary et al., 2015; Su et al., 2020). Previous research has shown that improved glucose metabolism is associated with an increased abundance of *Prevotella*, which may be linked to the capacity of *Prevotella* to protect against glucose intolerance (Kovatcheva-Datchary et al., 2015; Su et al., 2020). However, it's important to note that certain *Prevotella* species were associated with pro-inflammatory conditions, highlighting the need for species-specific analyses (Yang et al., 2021). The enrichment of *Prevotella* in Pg3G-treated mice suggests that Pg3G prevents T2DM in db/db mice, at least in part, by modulating the abundance of *Prevotella* (Kovatcheva-Datchary et al., 2015; Su et al., 2020). In addition, a comparative study explored the disparate biological effects of structurally distinct anthocyanins, indicating that acylated anthocyanins may exert more pronounced regulatory influences on energy metabolism, gut microbiota homeostasis, and inflammation in T2DM compared to non-acylated anthocyanins (Chen et al., 2023h), which could be explained partially by the higher stability of acylated anthocyanin (Yi et al., 2006; Zhao et al., 2017a), due to the acylation of the glycosyl moieties results in alterations to the chemical properties of anthocyanins (Jokioja et al., 2021; Zhao et al., 2017a). A crucial next step is to identify the specific bacterial enzymes responsible for the de-acylation and glycosylation of anthocyanins, which would open new avenues for targeted interventions (Chen et al., 2023h).

### 3.4 Neurodegeneration-related disturbances

Anthocyanins are also shown to protect the host from neurodegeneration, apoptosis, and oxidative stress in mice challenged by lipopolysaccharides (Khan et al., 2016). Many studies indicated that anthocyanins may have neuroprotective effects, which could help mitigate several conditions that affect the brain (Fig. S3), including impairments affecting cognition, neuroinflammation, abnormal amyloidogenesis, and apoptotic markers (Bayazid and Lim, 2024; Henriques et al., 2020). For example, the anthocyanin-rich extract of red cabbage cyanidin-3-diglucoside-5-glucoside-rich extract has been demonstrated to mitigate age-related cognitive decline by attenuating neuroinflammation and regulating the gut-brain axis, the specific effect on gut microbiota was increased prevalence of the Firmicutes phylum represented by the Clostridia class and Lachnospiraceae family, suggesting a potential shift in gut microbiota composition that favors neuroprotection (N. Zhang and Jing, 2023). While these findings are intriguing, it's important to note that many studies rely on animal models, and the direct translatability of these results to human physiology remains an area of ongoing investigation (Cao et al., 2024). The anthocyanins-induced increase in the relative abundance of the *Lactobacillus* genus may contribute to alleviating neuroinflammation, as supported by *in vitro* evidence demonstrating that anthocyanins promote the growth of *Lactobacillus* (Li et al., 2024; Peng et al., 2023). This provided evidence of a comprehensive cross-talk mechanism between anthocyanins and neuroinflammation in mice subjected to a high-fructose diet, which was mediated by ameliorating gut microbiota dysbiosis and preserving the integrity of the intestinal barrier (Peng et al., 2023). On the other hand, it has been proposed that the microbial-intestinal-brain axis may represent a hitherto unidentified mechanism through which anthocyanins exert a neuroprotective effect in the context of neurodegenerative diseases (NDs) (Zhong et al., 2023). Anthocyanins can achieve the therapeutic purpose of NDs through the regulation of intestinal microflora and certain metabolites, including PCA and vanillic acid (Zhong et al., 2023). The modulation of specific neurotransmitters by tryptophan metabolism and the maintenance of blood-brain barrier (BBB) permeability through butyrate production were demonstrated to play a preventive role in NDs (Zhong et al., 2023). This can be explained by the gut microbiota's ability to produce SCFAs and modulate the metabolism of bile acids and neurotransmitters like gamma-aminobutyric acid, serotonin, glutamate, and dopamine, influencing the gut-brain axis (Barrett et al., 2012; Zhong et al., 2023). Subsequent studies highlighted butyrate production by gut bacteria as a key factor in maintaining BBB integrity (Zhong et al., 2023). While some neurotransmitters produced in the gut cannot directly cross the BBB, the gut microbiota can convert them into functional neurotransmitters that can, such as dopamine and norepinephrine, thereby acting on the central nervous system (Barrett et al., 2012; Zhong et al., 2023). Collectively, anthocyanins may be posited as an invaluable therapeutic agent for combating or delaying the onset of Alzheimer's disease (Bayazid and Lim, 2024). In support of this, blueberry anthocyanin-rich extracts showed promise as a means of defending the human body against oxidative damage and neurodegeneration (Si et al., 2021). The modulation of the antioxidant profile observed may be attributed to the enhanced diversity of the gut microbiota, the stimulation of probiotics (specifically, *Bifidobacterium* and *Lactobacillus*), and SCFAs-producing bacteria (including *Roseburia*, *Faecalibaculum*, and *Parabacteroides*), which were increased by anthocyanins and their metabolites (Si et al., 2021). This has led to an improvement in the colon environment, which has been characterized by an increase in SCFAs, the restoration of colonic mucosa, and a reorganization of the microbial composition (Si et al., 2021). From a dietary trade-off perspective, it is suggested that anthocyanins from fruits and berries should be considered complementary supplements to ease neuro-disorder symptoms and alleviate chronic diseases of the central nervous system (Panchal and Brown, 2022).

### 3.5 Translational gap

Despite these findings, the precise roles of gut microbiota in health, their interactions, and metabolites in regulating gut microecology through anthocyanin intervention remain inadequately understood. Moreover, the disparate effects of individual anthocyanins and potential synergistic interactions with other phytochemical supplements, alongside the bioavailability of anthocyanins derived from various food matrices, merit further investigation (Vendrame and Klimis-Zacas, 2019), and the potential of anthocyanins with co-occurrence gut microbiota as natural therapeutic agents or therapeutic targets present a promising avenue for developing alternatives to conventional disease prevention and treatment strategies. Additionally, for the translational applications in humans, there is a need to establish the recommended therapeutic dose, frequency, and route of administration, along with developing more effective strategies to leverage its significant properties. Factors such as age, genetics, diet, and antibiotic use should be considered.

For example, although preclinical studies suggest that regular consumption of anthocyanin-rich fruits and vegetables can assist in the prevention of obesity and its associated comorbidities, as well as in weight management for both healthy and obese populations, further investigation is warranted to determine the optimal doses, methods of administration, and duration of treatment required to establish effective clinical protocols for anthocyanin-based therapies (Sivamaruthi et al., 2020). It's crucial to acknowledge that many *in vivo* studies rely on high concentrations of anthocyanins, which may not be readily achievable through dietary intake alone. Many *in vivo* studies also shown that dietary intake of anthocyanins protects against cardiovascular dysfunction and determines long-lasting cardioprotection in coronary heart disease (Dong et al., 2022; Trinei et al., 2022; Vendrame and Klimis-Zacas, 2019). These protective effects are linked to improvements in lipid profiles, vascular function, blood glucose control, and inflammation (Mohammadi et al., 2024; Zhao et al., 2024b). Despite the antioxidant, anti-inflammatory, and lipid-lowering properties associated with anthocyanin consumption being well-established, its effects on blood pressure regulation demonstrate variability, leading to inconclusive findings in human studies regarding its efficacy in preventing cardiovascular diseases (Grosso et al., 2022; Vendrame and Klimis-Zacas, 2019). For example, while some studies showed a correlation between anthocyanin intake and reduced risk of cardiovascular events (Krga and Milenkovic, 2019), others failed to demonstrate consistent blood pressure-lowering effects (Mattioli et al., 2020). This variability may stem from differences in study populations, anthocyanin sources, dosages, and the complex interplay of individual factors (Vendrame and Klimis-Zacas, 2019). A systematic review highlighted the influence of daily dosage, participant health status, and intervention duration on outcomes (Mohammadi et al., 2024). Concurrently, clinical evidence indicates that anthocyanins significantly affect vascular health in recipients receiving purified anthocyanin supplements or anthocyanin-rich whole berries, berry juices, berry powders, and purees (Mohammadi et al., 2024), additional research is indispensable to understand the specific types of molecules contributing to these effects, the variations in dosing between animal and human studies, and the timing of interventions associated with vascular benefits while also considering individual differences in anthocyanin absorption, gut microbiota composition, and metabolites (Grosso et al., 2022; Vendrame and Klimis-Zacas, 2019). Recent studies emphasize that the bioactivity and health-promoting effects of anthocyanins may differ from those of their metabolites, highlighting the need for further research on the bioavailability of metabolites in isolation and mixtures (Festa et al., 2023). Further investigation is warranted into the interactions between anthocyanins and gut microbiota, particularly the elucidation of complex metabolic and immune pathways (Wang et al., 2024d). For example, anthocyanins can modulate gut microbial populations, increasing beneficial bacteria like *Lactobacillus* and *Bifidobacterium* (Igwe et al., 2019; Wang et al., 2025e), while inhibiting harmful bacteria (Wang et al., 2025e). Understanding how genetic factors and microbiota enterotypes influence anthocyanin metabolism is also essential (Vendrame and Klimis-Zacas, 2019). Finally, research should explore the potential for anthocyanins and their metabolites to transcriptionally reprogram critical cells, offering new avenues for therapeutic intervention (Zhao et al., 2024b). By addressing these gaps in knowledge, we can better harness the health-promoting potential of anthocyanins and develop targeted dietary strategies to improve vascular and overall health.

## 4 Exploring anthocyanin bioavailability: implications for gut microbiota and prebiotic interactions

Although anthocyanin consumption is linked to reduced chronic disease risks, their low bioavailability and inter-individual variability in clinical responses remain key challenges (Di Lorenzo et al., 2021; Fernandes et al., 2014; Teng and Chen, 2019). A significant fraction of ingested anthocyanins escapes absorption in the upper gastrointestinal tract and reaches the colon, where gut microbiota metabolize them via enzymes such as β-d-glucosidase and β-d-glucuronidase (Liang et al., 2023; Tian et al., 2019a). This microbial biotransformation generates bioactive metabolites (e.g., phenolic acids) with enhanced absorption potential, thereby indirectly improving systemic bioavailability and modulating host health (Saini et al., 2024; Singh et al., 2019; Teng and Chen, 2019).

Emerging strategies to optimize anthocyanin delivery include microbial biosynthesis and gene-editing technologies, which enable scalable production and structural modifications to enhance stability (Alappat and Alappat, 2020; Ayvaz et al., 2022; Lila, 2004; Xie et al., 2018). Noteworthy, the bioavailability of compounds produced via these approaches—particularly microbially biosynthesized anthocyanins and gene-edited variants—needs further investigation. Together, these advances underscore the dual role of gut microbiota: as a determinant of anthocyanin metabolic fate and a target for precision nutrition strategies.

### 4.1 *In vitro* case study on physical-chemical dependent anthocyanins bioavailability and characteristics

Physical-chemical characteristics as affected by physical and chemical treatments, including high-pressure processing, ultrasound, polysaccharide addition, and self-assembly, can modulate the interactions between proteins and the subclasses of dietary polyphenols, thus enhancing the functionality of proteins and bioactives' quality, safety, and preservation (Kim et al., 2024). Dietary proteins, such as whey protein, was shown to interact with anthocyanins, enhancing the color, stability, antioxidant capacity, and bioavailability of the resulting complex (Ma et al., 2023b; Ren et al., 2021; Ren and Monica Giusti, 2021; Wang et al., 2023f). These interactions are primarily driven by hydrophobic forces, though electrostatic interactions and hydrogen bonding also contribute (Ren et al., 2021). Beyond whey protein, anthocyanins' interactions with proteins from various food sources, including soy, rice, and pea, exhibit unique characteristics, significantly influencing the stability and bioaccessibility of anthocyanins (Ahmadi et al., 2023; Li et al., 2021e; Li et al., 2020f). Studies on soy protein isolate reveal that both non-covalent and covalent interactions with anthocyanins can occur, impacting the functional and conformational properties of the protein (Chen et al., 2019i; Sui et al., 2018). For instance, preheating soy protein isolate can modify its secondary structure, altering its binding affinity for anthocyanins and modulating the protective effect on anthocyanin stability (Chen et al., 2019i; Ren et al., 2021). Similarly, rice proteins interact with anthocyanins, affecting the protein's structural and functional attributes, such as foaming capacity (Li et al., 2020f; Wang et al., 2023f). The type of interaction between protein/polysaccharide and anthocyanins mainly depends on their structures, and the noncovalent interaction between them is the key intermolecular force that increases the stability of anthocyanins (Zang et al., 2022). Pea protein, increasingly used in food formulations, also demonstrates interactions with anthocyanins, influencing bioaccessibility and stability (Ahmadi et al., 2023; Cesa et al., 2025). In a comprehensive investigation of the interactions between anthocyanins and whey protein isolate, as well as bovine serum albumin, it was demonstrated that these proteins confer a protective effect on the stability of anthocyanins while simultaneously enhancing the solubility, foaming, and emulsifying properties of the proteins due to the interactions with anthocyanins (Zang et al., 2021). Furthermore, it is important to note that the structural diversity among different anthocyanins leads to variations in their binding forces and affinities for specific proteins (Ren et al., 2021). The pretreatment or processing of native proteins can also significantly influence these binding affinities, highlighting the necessity for further investigation to elucidate the distinct interaction mechanisms on a case-by-case basis (Lin et al., 2023; Ren et al., 2021). Understanding how various molecules, such as fatty acids, glucose, metal ions, and metabolites, influence the interactions between proteins and anthocyanins presents a significant challenge (Lin et al., 2023). The formation of complexes between anthocyanins and proteins may be adversely affected by the competitive binding forces exerted by these molecules on specific proteins (Lin et al., 2023), thereby impacting the stability of the anthocyanin-protein complexes and their subsequent digestive fates.

#### 4.1.1 Cell culture model on innate structure-dependent anthocyanins bioavailability

The Caco-2 cell line, an established model of human intestinal epithelial cells derived from colorectal carcinoma, has proven to be a reliable supplementary approach to animal studies for predicting the intestinal absorption of anthocyanins (Kamiloglu et al., 2015). Research examining the absorption of anthocyanins by Caco-2 cells has consistently demonstrated minimal uptake of these compounds. However, it is important to consider that the bioavailability of anthocyanins may be significantly underestimated; the metabolites generated during digestion could play a crucial role in mediating the health benefits attributed to anthocyanins (Kamiloglu et al., 2015; Lila et al., 2016).

The cellular uptake of anthocyanins was concentration-dependent and affected by their structural features, particularly ring B substitutions (Cahyana et al., 2022). Experimental findings demonstrate that glucose-based anthocyanins exhibit greater bioavailability than galactose-based counterparts, and that increased hydroxylation (particularly on ring B) correlates with reduced bioavailability, while methoxylation enhances stability and uptake (Husain et al., 2022; Yi et al., 2006). This is attributed to two key factors: 1. Methoxyl groups (-OCH_3_) protect anthocyanins from nucleophilic attack by water or enzymatic degradation (e.g., via catechol-O-methyltransferase), whereas hydroxyl groups (-OH) increase susceptibility to oxidation and pH-dependent degradation (Husain et al., 2022; Ichiyanagi et al., 2006; Sandoval-Ramírez et al., 2018; Talavéra et al., 2005; Yi et al., 2006); 2. Hydroxyl groups enhance hydrophilicity, limiting passive diffusion across intestinal membranes, while methoxyl groups increase lipophilicity, favoring uptake via transcellular transport (Cahyana et al., 2022; Husain et al., 2022). Specifically, the average efficiency of anthocyanins transport was approximately 3–4 %, while delphinidin-3-O-glucoside (Dp3G, containing two hydroxyls at the R_1_ and R_2_ substitution position of ring B of the anthocyanidin structure, respectively, see Fig. 1) was reported to be less than 1 % (Husain et al., 2022; Ichiyanagi et al., 2006; Sandoval-Ramírez et al., 2018; Yi et al., 2006). In particular, at low concentrations, strawberry or red grape concentrate extracted Cy3G (containing one hydroxyl group at the R_1_ substitution position of ring B of the anthocyanidin structure, see Fig. 1.), cellular uptake was higher than pelargonidin-3-O-glucoside (Pg3G, containing no hydroxyl group substitution position of ring B of the anthocyanidin structure, see Fig. 1.) uptake in the study (Cahyana et al., 2022). The increase in anthocyanin uptake, concomitant with a decrease in pH, suggests a potential correlation between lowered pH levels and enhanced anthocyanin stability, possibly mediated by proton co-transporters (Cahyana et al., 2022). The presence of glucose diminished the uptake of anthocyanins, suggesting a potential role for facilitative glucose transporters in mediating anthocyanin absorption by Caco-2 cells (Cahyana et al., 2022). In conclusion, the variations observed in the absorption, degradation, distribution, and excretion of anthocyanins are primarily influenced by the specific structures of their glycosides and aglycones (Gui et al., 2023).

#### 4.1.2 Multi-component-based *in vitro* simulated digestion model and considerations

Food component-based *in vitro* digestion models are widely utilized to investigate the stability and bioavailability of polyphenolics, with a particular emphasis on the subclass of anthocyanins. The interaction between anthocyanins and food components, including proteins and polysaccharides, significantly influences the chemical stability of anthocyanins throughout food processing and storage (Zang et al., 2022). In the present study, researchers employed model systems to demonstrate that the variations in the chemical structures of polyphenolic compounds lead to differing stability profiles and capacities for interaction with macromolecules (Stübler et al., 2021). Moreover, these interactions, along with the overall stability of polyphenolic compounds, are profoundly affected by both traditional and innovative processing technologies and formulations. These changes in stability can subsequently culminate in a diverse range of effects on bioavailability (Stübler et al., 2021). For example, Pg3G, which represents the predominant anthocyanin component in strawberries, contributes to the red color and antioxidant properties of strawberries (Zou et al., 2018). Thermal and high-pressure processing treatment significantly altered Pg3G interaction with α-lactalbumin under different pH values; such interaction may provide insights into the impact of food processing conditions on the stabilization strategies for anthocyanins (Tian et al., 2022c). Additionally, *in vitro* polyphenol bioavailability is studied using a multi-component model. Thermal processing often induces degradation (reducing stability), which inevitably leads to decreased polyphenol bioavailability, while high-pressure processing mostly preserves the original polyphenol chemical integrity (stability) and consequently its bioavailability profile (Eran Nagar et al., 2021). Moreover, processing and formulation significantly influence the stability and bioavailability of dietary polyphenols during storage, according to a study that examined the impact of various preservation technologies on strawberry polyphenols, as well as the effects of incorporating a relatively protein-rich kale juice, highlighting the intricate relationship between these factors and polyphenol stability over time and its consequences for bioavailability (Stübler et al., 2019). Similarly, plant-based canola protein and polyphenol multi-component interaction models are also proven to enhance polyphenol stability and correlate with mitigated losses in antioxidant levels of strawberry polyphenols during storage (Stübler et al., 2021).

Critically, it was found that digestion conditions, as tested *in vitro*, have the greatest impact on the dietary polyphenols’ antioxidant properties (Jiao et al., 2018) and are often linked with altered bioavailability. More recently, the study found that the dissolved oxygen and the presence of bile compounds in the native digestive fluid environment can largely impact the bioavailability of dietary polyphenols in the intestinal digestion phase, for which less attentions were paid (Eran Nagar et al., 2023). Therefore, more reasonably, we may speculate that such interactions exist between these subclasses of dietary polyphenols and the physiological digestive fluid, which contributes significantly to the decreased bioavailability profile of these bioactive compounds *in vivo*.

In conclusion, efforts made to elucidate the chemical interactions between anthocyanins and macromolecules, the impact of food processing and storage conditions on stability, and simulated gastrointestinal digestions are all critical factors for a better understanding of anthocyanins’ bioavailability *in vitro*. The stability of the compound, particularly its resistance to degradation, is a primary determinant influencing its potential bioavailability.

### 4.2 *In vivo* case study on tissue and source-dependent anthocyanins bioavailability and implications

Extensive animal and clinical studies demonstrated that anthocyanin-rich foods or extracts yield only minimal concentrations of anthocyanins or their predicted metabolites in plasma following standard blood sampling, suggesting a low bioavailability (Lila et al., 2016; Wu et al., 2023). Furthermore, while *in vitro* models and cell lines often produce various metabolites, the assessment of these metabolites *in vivo* remains limited. Additionally, current evidence regarding the distribution of metabolites to target sites is insufficient (Rai and Tzima, 2021). Given the limitations of *in vitro* experiments in accurately replicating *in vivo* conditions, conducting more human clinical studies with larger sample sizes on anthocyanin bioavailability is becoming imperative (Lin et al., 2023; Wu et al., 2023).

Following the ingestion of anthocyanins, these compounds undergo partial hydrolysis by the oral microbiota, resulting in the formation of aglycones, which are subsequently conjugated by glucuronidase within the oral cavity (Gui et al., 2023). In the stomach, anthocyanins are absorbed intact, without deglycosylation, mediated by specific transporters such as sodium-dependent glucose co-transporter 1 (SGLT1) and facilitative glucose transporters 1 (Fang, 2014; Gui et al., 2023). The predominant mechanism for small intestinal absorption of anthocyanins involves SGLT1 and facilitative glucose transporters 2 (GLUT2) as well as other glucose transporters, whereas further studies demonstrating the genetic or chemical inhibition of SGLT1 or GLUT2 underscore the crucial role of these transporters in facilitating the absorption of anthocyanins across enterocytes, thereby enabling their bioactive properties (Solverson, 2020), and with the most absorption and metabolism occurring in the distal lower bowel of the gastrointestinal tract (Fang, 2014; Liang et al., 2023). Interconversion may occur due to the activity of enzymes, particularly catechol-O-methyltransferase. In the lower intestine, there is significant microbial catabolism of anthocyanins, which is further modified by phase I and phase II metabolic enzymes in the cells, after which they are absorbed (Gui et al., 2023; Mattioli et al., 2020). The process leads to the formation of hybrid microbial-host metabolites, which are released into the bloodstream through the gastrovascular cavity, subsequently entering the enterohepatic circulation. This mechanism significantly enhances the bioavailability of anthocyanins (Gui et al., 2023; Mattioli et al., 2020). For instance, the pharmacokinetics of Cy3G and its metabolites in humans were thoroughly investigated, employing isotope-labeling techniques (De Ferrars et al., 2014). Thirteen metabolites derived from PCA and one metabolite derived from phloroglucinaldehyde were identified as degradation products of Cy3G; among these metabolites, hippuric acid and ferulic acid were recognized as the principal phenolic metabolites (De Ferrars et al., 2014). Recent studies increasingly demonstrated that enterohepatic recycling contributes to an extended residence time of anthocyanin metabolites. These metabolites, which maintain the distinctive C6-C3-C6 flavonoid backbone structure, provide substantial support for the assertion that anthocyanins possess greater bioavailability than previously posited (Gui et al., 2023; Lila et al., 2016).

On the other hand, the biodistribution and structure of anthocyanins upon absorption were studied and can be tissue and source-dependent. For example, Cy3G was detected in the rat plasma immediately after oral administration of a Cy3G-containing diet (Tsuda et al., 2000). Additionally, PCA, which appears to be produced by the degradation of cyanidin, was also detected in the plasma. Whereas in the liver and kidneys, Cy3G undergoes metabolism to yield a methylated derivative (Tsuda et al., 2000). Another study examined anthocyanins metabolism and distribution in the digestive tract organs, kidney, and a target tissue in rats fed with a blackberry anthocyanin-enriched diet, indicating a metabolic pathway of anthocyanins with tissue-dependent enzymatic conversions (methylation and glucurono-conjugation) and demonstrated that anthocyanins could enter the brain (Talavéra et al., 2005). He et al. investigated the impact of chemical structure on anthocyanin absorption and metabolism in rats, suggesting that tissue-bound anthocyanins may help to explain the low plasma availability of anthocyanins (He et al., 2006). Furthermore, the type and number of anthocyanin glycosylations affected the absorption remarkably (He et al., 2006). In a conducted study, the physiological effects and tissue distribution of bilberry anthocyanins were examined; the findings indicated that following the oral administration of bilberry extract, both unmodified and methylated anthocyanins were identified in the plasma of mice and exhibited specific distribution patterns within key organs, notably the liver, kidney, and testis (Hiroyuki et al., 2009). Malvidin glycosides were the most prevalent bilberry anthocyanins distributed in tissues (Hiroyuki et al., 2009). Del Bò et al. investigated the effect of wild blueberry diet consumption on anthocyanin distribution and metabolism in Sprague-Dawley (SD) rats and revealed that the extent of anthocyanin metabolism and subsequent excretion was notably influenced by the duration of the dietary intervention (Del Bò et al., 2009). Furthermore, urinary hippuric acid content may serve as a potential biomarker of anthocyanin absorption and metabolism in SD rats under experimental settings (Del Bò et al., 2009). Kay et al. suggested that a significant proportion of intestinal metabolites of anthocyanins are likely to be conjugates of their degradation products (Kay et al., 2009). The rapid transit of oral bioactive molecules through the stomach, coupled with their limited permeability and solubility in the gastrointestinal tract, renders anthocyanins particularly vulnerable to degradation influenced by environmental factors and the conditions present during digestion (Teng et al., 2023). Consequently, it is necessary to investigate the phenolic and aldehyde degradation products, along with their respective metabolites, to ascertain the bioavailability of anthocyanins *in vivo*.

A variety of analytical techniques were established for the qualitative and quantitative analysis of phenolic metabolites in anthocyanin-rich fruits, utilizing both *in vitro* and *in vivo* methodologies (Rai and Tzima, 2021). Key among these techniques are liquid chromatography and mass spectrometry, along with other hyphenated methods. These advanced analytical tools were proven invaluable for the detailed characterization and profiling of metabolites, offering substantial evidence of their properties, formation mechanisms, and health benefits (Alappat and Alappat, 2020; Khoo et al., 2017; Rai and Tzima, 2021).

## 5 The structural and technological requirements for optimizing the bioavailability and health benefits of anthocyanins

Given the comprehensive effect of anthocyanins is of paramount importance in promoting growth and health, including the promotion of gut microbiota and immunity, enormous efforts were made to improve anthocyanin's bioavailability such as from the perspectives of the solubility and efficient transport of dietary anthocyanins throughout the gastrointestinal tract into the targeted intestinal regions, thus allowing for maximizing the health benefits of anthocyanin's administration (Guo and Shahidi, 2024; Saini et al., 2024).

Modifying the structure of these compounds may serve as a vital strategy to enhance their bioavailability and associated health benefits. For instance, fructo-oligosaccharides (FOS) were shown to augment the antioxidant activity of anthocyanins derived from red radish (ARR) by improving their bioavailability (Li et al., 2023g). This enhancement occurs through FOS-mediated modulation of gut microbiota, which promotes the hydrolysis of complex pelargonidin glucosides, ultimately yielding pelargonidin and simpler glucosides easily absorbed into the bloodstream (Li et al., 2023g). These findings suggest that the concurrent consumption of FOS and ARR may provide an effective approach to enhance the bioactivity of anthocyanins sourced from red radish (Li et al., 2023g). Another recently reported approach involves utilizing protein carriers to bind anthocyanins, which has shown a significant enhancement in their bioactivity and bioavailability compared to anthocyanins delivered without a protein carrier (Lila et al., 2022; Ren et al., 2021; Wu et al., 2023), this enhancement occurs because the phytoactive anthocyanins are safeguarded within the edible protein matrix as they transit through the gastrointestinal tract, enabling intact, non-degraded molecules to reach the colon for catabolism at the gut microbiome level (Lila et al., 2022). Food compounds, notably α-casein, have the potential to enhance the absorption of blueberry anthocyanins and their metabolites into the bloodstream in rat models (Lang et al., 2021). Additionally, molecular docking studies suggest that anthocyanins can traverse the structural cavity of α-casein and engage with its amino acid residues, a finding that aligns with the observed increase in anthocyanin bioavailability (Lang et al., 2021). α-casein also effectively controlled the excretion of blueberry anthocyanins and their metabolites, thus improving bioavailability (Lang et al., 2023). This was evidenced by increased absorption of anthocyanins in the bloodstream, reduced levels of bioactive compounds of the anthocyanin family in urine, such as Cy3G, Mv3G, Dp3G, and metabolite content in feces, such as syringic acid, ferulic acid, and gallic acid (Lang et al., 2023). Given that most anthocyanins are unarguably metabolized by the gut microbiome, delivery of an intact form of protein-anthocyanin complexes to the colonic microbiota is preferable, considering protein metabolic fate during early digestion in the gastrointestinal tract (Lila et al., 2022; Singh et al., 2019; Wu et al., 2023). However, research focusing on the interaction of food components with anthocyanins is currently limited by a lack of structure-specific selection, and the stabilization methods employed are not optimal (Li et al., 2024a). This inadequacy hampers the attainment of precise delivery and targeted release of anthocyanins *in vivo* (Zang et al., 2022).

To overcome these challenges, technological solutions such as microencapsulation were developed and implemented for the formulation of anthocyanin-enriched food products (Teng et al., 2023). As for smart formulation design for improved bioavailability profile *in vivo*, bioactive delivery systems based on proteins, carbohydrates, lipids, and a mix of these macromolecules are widely developed (Ijinu et al., 2023; Singh et al., 2019). Furthermore, the novelty in the application of nanoencapsulation based on polysaccharides, proteins, and lipids can facilitate the protection of anthocyanins throughout the gastrointestinal tract, enabling their controlled release (Burillo et al., 2022; Teng et al., 2023). In a mouse model of obesity induced by a high-fat diet, oral administration of anthocyanins-LNP (liver-targeted nanoparticle) demonstrated better efficacy in reducing body weight, mitigating liver injury, and reducing lipid droplet accumulation in the liver compared to anthocyanins alone (Chen et al., 2024c). Furthermore, anthocyanins-LNP demonstrated a significant capacity to restore the gut microbiota balance disrupted by a high-fat diet (Chen et al., 2024c). The implementation of nanotechnology for intelligent protection, controlled delivery, and tissue-specific targeted delivery can serve to diminish the impact of microbiota on the biotransformation of anthocyanins (Burillo et al., 2022; Singh et al., 2019). This represents a more effective process of absorption of intact forms and preserves their biological effects, including antioxidant activity and certain metabolic modulations (Burillo et al., 2022). While the studies are successful in the nanoencapsulation of anthocyanins, there is still a paucity of research in the *in vivo* domain, particularly in animal and humanized models.

However, these encapsulation techniques have disadvantages in terms of relatively weak protective effects on anthocyanins against external environments and color impairment (Tan et al., 2021b). In addition, the influence of food components and delivery systems on anthocyanins' processing and digestive stability warrants significant examination (Tan et al., 2021b; Zang et al., 2022). Importantly, when considering the bioavailability and health benefits of bioactive compounds, the type of processing technologies and formulations can significantly influence the bioavailability profile of many health-promoting compounds. For example, the high hydrostatic pressure-treated Cy3G-blueberry pectin complexes exhibited better anti-inflammatory effects on DSS-induced colitis mice (Tan et al., 2022b). Conclusionally, it is essential to gain a more profound comprehension of the potential impact of biological interactions, encompassing microbiota, critical cells, enzymes, and certain biological systems, on anthocyanins’ bioavailability, a prerequisite for realizing the appreciable health benefits of these compounds.

## 6 Conclusion and perspective

**Fig. 4:**
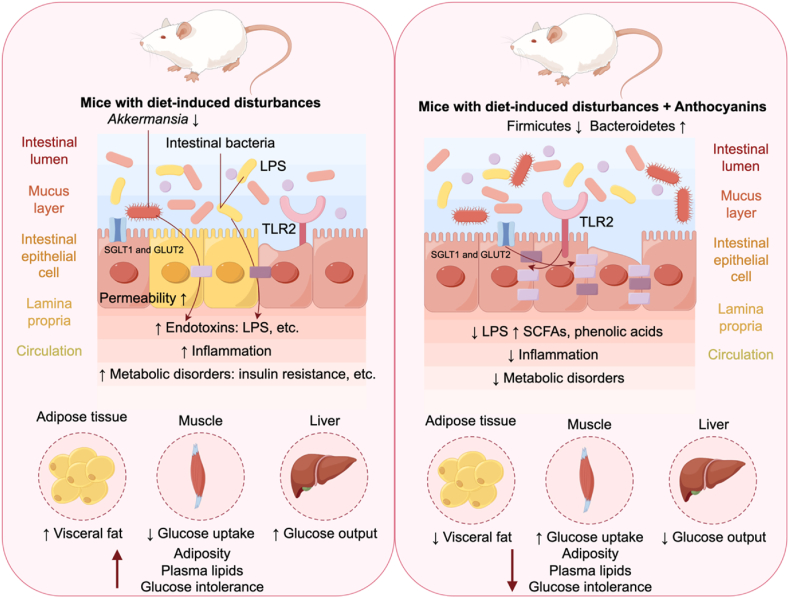
Schematic showing the potential biological outcomes of anthocyanins intake on gut microecology and peripheral tissues in common diet-induced disturbances. Mice with diet-induced disturbances were associated with gut dysbiosis as evidenced by, for example, a decreased population of the *Akkermansia* genus (Gu et al., 2023; Su et al., 2020), which could lead to increased levels of endotoxins such as lipopolysaccharides (LPS) and other inflammatory markers (Chen et al., 2022g; Sun et al., 2022) mediated by, for example, toll-like receptor 2 (TLR2) immune signaling pathways, and manifestation of metabolic disorders such as insulin resistance, etc. (Xie et al., 2018). As a result, the uptake of anthocyanins may reverse these changes, specifically in glucose and lipid metabolism (Chen et al., 2022g; Sun et al., 2022). The predominant mechanism for small intestinal absorption of anthocyanins involves sodium-dependent glucose co-transporter 1 (SGLT1) and facilitative glucose transporters 2 (GLUT2), as well as other glucose transporters (Fang, 2014; Liang et al., 2023). The potential biological outcomes may include decreased glucose uptake in the muscle, increased visceral fat storage in adipose tissue, and glucose output in the liver caused by the diet-induced disturbances (Solverson, 2020). Created by http://www.figdraw.com/#/.

The interplay between anthocyanin bioavailability, gut microbiota dynamics, and health benefits forms a key relationship in nutritional science. While current research acknowledges the role of gut microbiota in anthocyanin metabolism, the precise mechanisms governing the interactions between phenolic metabolites, gut microbiota, mucosal immune responses, and metabolic signaling pathways remain largely unknown. This is particularly true across diverse health and disease states (Fig. 4).

Emerging evidence suggests that anthocyanins may act synergistically with other dietary components, including vitamins and minerals, potentially functioning as dietary prebiotics that enhance metabolic health and bolster immune function. The bioavailability of anthocyanins is significantly affected by food components and processing techniques. To fully understand the impact of anthocyanins, rigorous investigations are needed to determine how food processing and co-ingestion of dietary supplements affect health outcomes related to anthocyanin consumption, host-microbiota interactions, the production of gut metabolites and standardize the reporting of anthocyanin profiles in food-based interventions and explore the impact of food matrices on the observed effects to better understand the translational potential of these findings. A key question remains whether the health effects observed are due to the parent anthocyanins or their metabolites, and what factors most significantly influence bioavailability, particularly concerning the gut microbiota.

Future research should focus on several key areas to address these gaps and controversies. First, standardized and validated methods for assessing anthocyanin bioavailability *in vivo* are crucial, considering the limitations of current *in vitro* models. Second, multi-omics approaches (genomics, proteomics, metabolomics, and transcriptomics) should be employed to elucidate the complex interactions between anthocyanins, gut microbiota, and host tissues. This includes identifying specific microbial species responsible for anthocyanin metabolism and characterizing the resulting metabolites with potential bioactivity. Third, more research is needed to investigate the impact of inter-individual variability in gut microbiota composition on anthocyanin metabolism and bioavailability. Specific strategies to improve anthocyanin bioavailability and optimize their interaction with the gut microbiota should be explored. These include encapsulation techniques: employing nanoemulsions or liposomes to protect anthocyanins from degradation in the upper gastrointestinal tract; co-administration with prebiotics: combining anthocyanins with established prebiotics like inulin or FOS to selectively promote the growth of beneficial bacteria that can enhance anthocyanin metabolism; protein-binding approaches: investigating the use of protein carriers to improve anthocyanin bioaccessibility and bioavailability; structural modifications: chemically modifying anthocyanins to enhance their stability and less prone to degradation.

Finally, the identification of functional and bioactive compounds is critical, especially in the context of high-throughput discovery methodologies. Advances in digital twin technologies and various omics disciplines substantially improved our ability to discover these compounds within food matrices. Integrating high-throughput screening techniques and computational prediction models facilitates innovative methodologies for identifying and designing functional bioactive compounds. Research efforts should emphasize high-throughput approaches, computational strategies, informatics-based methods, and both *in vitro* and *in vivo* bioactivity assays, highlighting novel technologies that elucidate the molecular mechanisms underlying health benefits and determine whether they are attributable to or independent of the context of disorders. Such advancements represent a significant convergence of technology and food science aimed at enhancing gut health and nutrition. By addressing these research gaps and exploring innovative strategies, we can unlock the full potential of anthocyanins to promote human health.

## CRediT authorship contribution statement

**Fuqing Gao:** Conceptualization, Methodology, Investigation, Formal analysis, Visualization, Writing – original draft. **Peiqing Yang:** Conceptualization, Writing – review & editing. **Wenxin Wang:** Conceptualization, Writing – review & editing. **Kewen Wang:** Conceptualization, Visualization, Writing – review & editing. **Liang Zhao:** Conceptualization, Writing – review & editing, Funding acquisition. **Yongtao Wang:** Conceptualization, Writing – review & editing, Funding acquisition. **Xiaojun Liao:** Conceptualization, Visualization, Writing – review & editing, Supervision, Funding acquisition.

## Declaration of competing interest

The authors declare that they have no known competing financial interests or personal relationships that could have appeared to influence the work reported in this paper.

## Data Availability

Data will be made available on request.
