# Supplementary material for: Unveiling the multifaceted roles of anthocyanins: a review of their bioavailability, impacts on gut and system health, and industrial implications
Source: Curr Res Food Sci. 2025 Jul 9;11:101137. doi: 10.1016/j.crfs.2025.101137 (PMC12284573; doi:10.1016/j.crfs.2025.101137)

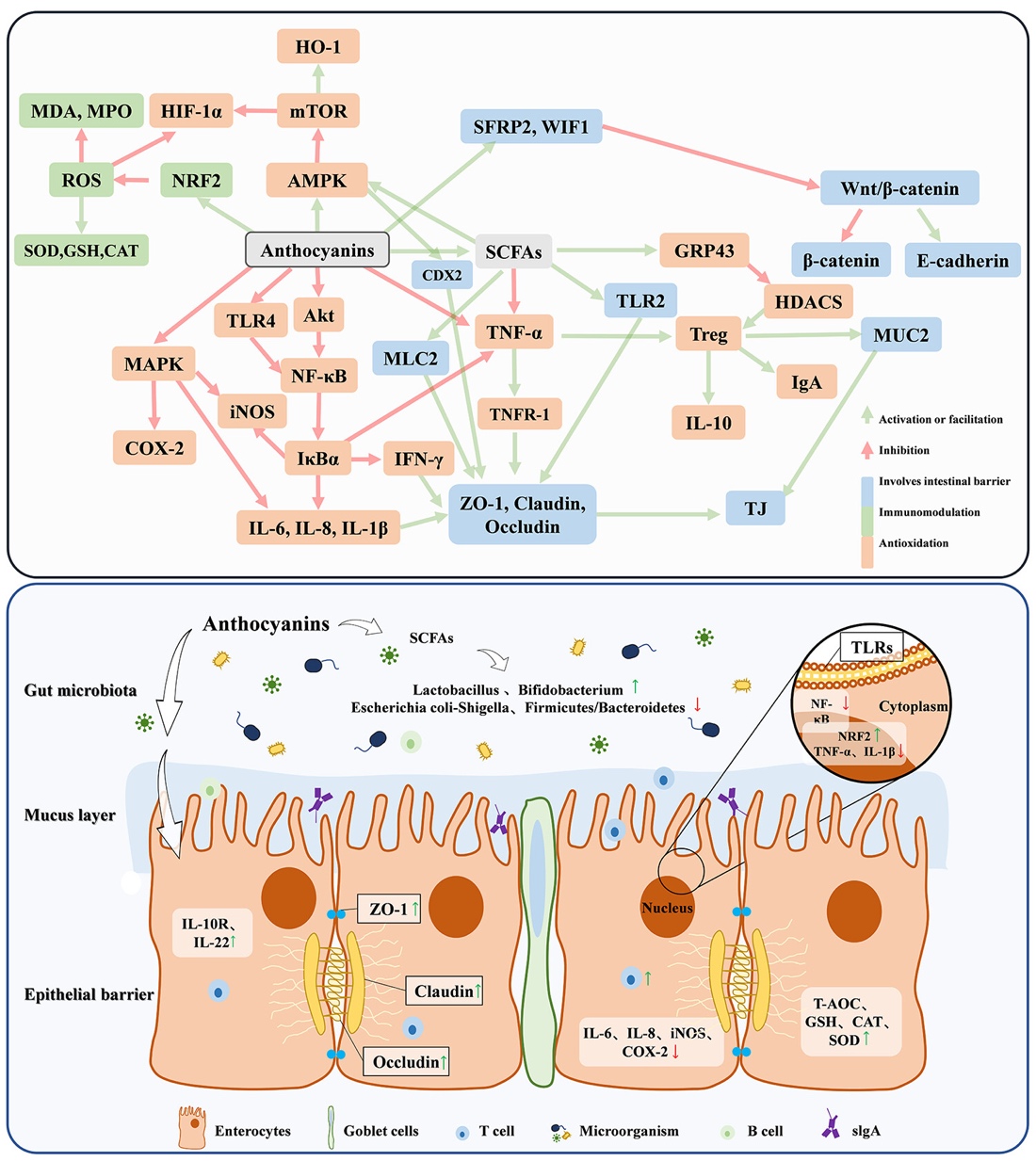
**Fig. S1. Schematic showing the role of anthocyanins and their metabolites in alleviating intestinal diseases. Source:** (Liu et al., 2024a)


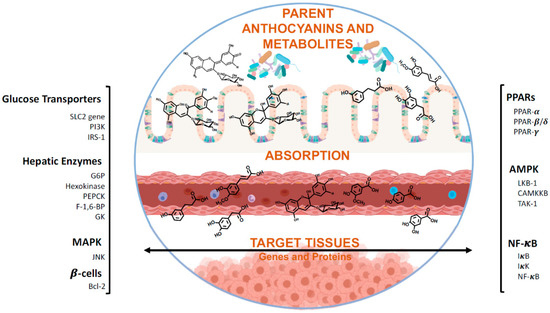
**Fig. S2. Schematic showing the role of anthocyanins and their metabolites with antidiabetic activity. Source:** (Oliveira et al., 2020)

**Fig. S3. Schematic showing the role of anthocyanins and their metabolites with anti-neuroinflammatory activity. Source:** (Henriques et al., 2020)


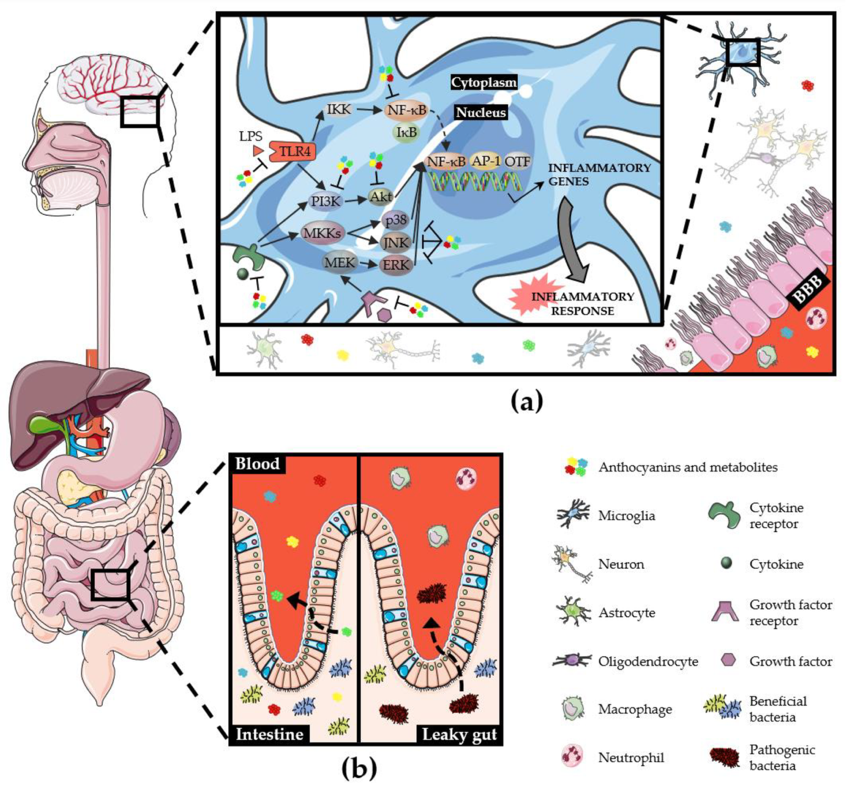

Supplement: Multimedia component 1 [file mmc1.docx]
